# Analysis of the Green Development Effects of High-Speed Railways Based on Eco-Efficiency: Evidence from Multisource Remote Sensing and Statistical Data of Urban Agglomerations in the Middle Reaches of the Yangtze River, China

**DOI:** 10.3390/ijerph192416431

**Published:** 2022-12-07

**Authors:** Xiangjing Zeng, Yong Ma, Jie Ren, Biao He

**Affiliations:** 1 School of Tourism, Hainan University, Haikou 570228, China; 2 Hainan Provincial Tourism Research Base, Haikou 570228, China; 3 Tourism Development and Management Research Center, Key Research Institute of Humanities & Social Sciences of Hubei Provincial Department of Education, Wuhan 430062, China; 4 Tourism Development Institute, Hubei University, Wuhan 430062, China; 5 School of Business Administration, Zhongnan University of Economics and Law, Wuhan 430073, China

**Keywords:** eco-efficiency, high-speed railways, multisource remote sensing data, spatial effects, urban agglomerations, middle reaches of the Yangtze River, county

## Abstract

As part of the modern transport infrastructure, high-speed railways (HSRs) have been considered an important factor affecting eco-efficiency (EE). This study used multisource remote sensing and statistical data from 185 counties representing urban agglomerations in the middle reaches of the Yangtze River (UAMRYR) in China from 2009 to 2018. The study integrated ArcGIS analysis, the Super-SBM (super slack-based measure) model, and the DSPDM (dynamic spatial panel Durbin model) to explore the spatial effects of HSRs on EE. The results showed that the coordinates of the interannual centers of gravity for EE and HSRs both fell in the same county, possessing similar parameter values for the standard deviation elliptical, a negative spatial mismatch index, and obvious spatial mismatch characteristics. In different spatially dislocated areas, the spatial effects of HSRs on EE are variable. Overall, the short-term effects are more intense than the long-term effects, and both the long-term and short-term effects are dominated by the effects of spatial spillover. A new perspective is proposed to explore the green development effects of HSRs, with a view to providing policy implications for the enhancement of EE and the planning of HSRs.

## 1. Introduction

Driven by the twin goals of ‘Carbon Peaking’ and ‘Carbon Neutrality’, green transformation has become the dominant mode of national production. In China, the focus of economic development is shifting from ‘high speed’ to ‘high quality’. In 2022, in the report of the 20th National Congress of the Communist Party of China, General Secretary Xi Jinping emphasized ‘the need to firmly establish and practice the Two Mountains Theory that lucid waters and lush mountains are invaluable assets, and to plan development at the height of the harmonious coexistence of man and nature.’ This indicates that green development is the main way forward and the driving force for China’s high-quality development and the construction of a beautiful China. It has become the new direction for harmonious development of economy and environment in China. This context makes the study of the Chinese case particularly revealing for solving the economic and environmental problems of the world.

It is necessary to assess scientifically the economic and environmental effects of HSRs in China. As a prominent aspect of sustainability, EE is an important indicator of the assessment system. It was proposed by Schaltegger and Sturm [1] that after being promoted by international organizations such as the World Business Council for Sustainable Development (WBCSD), the connotative expression of EE has been recognized as the ratio of the value of products and services to the ecological load. The balance between increment of economic output and reduction of negative environmental output has been emphasized. Studies have shown that the flows of inter-regional production factors are significantly influenced by transportation, which affects EE to a certain degree [2]. Naturally, EE is also affected by HSRs. Therefore, if part of the freight resources can be released by supply-oriented HSRs, in consideration of efficiency and sustainability, it can provide an important driving force to improve EE.

In academia, the economic effects, environmental effects, innovation effects, and industrial effects of HSRs have been discussed. The content of current research is mainly focused on economic and environmental effects. In terms of their economic effects, HSRs promote economic growth by reshaping economic patterns [3,4,5], improving spatial structure [6], increasing efficiency of resource allocation [7], and generating positive spillover effects [8]. Others believe that HSRs can strengthen the core periphery effects [9], central periphery effects [10,11], and siphon effects [12]. Additionally, it has been confirmed that the construction of HSRs involves huge costs to obtain relative benefits [13]. However, there is no consensus conclusion on the environmental effects of HSRs. Some studies have demonstrated the emission-reduction effects of HSRs including fossil fuel use [14,15,16], haze reduction effects [17,18,19], and industrial pollution emissions [20,21], while others after assessing the energy consumption of HSR construction have argued that HSRs have negative impacts [22,23,24]. In terms of theories, the life-cycle model was frequently used in the early stages of research. With ongoing research, the dual differences-in-differences (DID) model has gradually been introduced, and heterogeneity has been dentified mainly according to geographical location, city size, and industrial activity.

There has been abundant research exploring the effects of HSRs, and these previous studies provide an excellent basis for the current paper. Nevertheless, the following research questions have received comparatively less attention: Whether the indicators for the integrated evaluation of economic effects and environmental effects are influenced by HSRs? Whether HSRs have green development effects? What are the characteristics of the nature of these effects? Are there spatial heterogeneity and path-dependent characteristics? All these questions require further exploration. To respond to the above-stated questions, it is necessary to (1) enrich the research on the green development effect of HSRs, such as EE; (2) further investigate the spatial spillover effects of HSRs on EE; (3) introduce the SPDM and the DSPDM, and (4) use a reliable basis for heterogeneity studies.

To fill these gaps, taking EE as an example, this study is a preliminary attempt to explore the green development effects of HSRs. In the current research, taking the UAMRYR as a study case, multi-source remote sensing data were combined to construct a more comprehensive index system of EE. Moreover, the values of EE from 2009–2018 were measured by a more accurate model, namely, the Super-SBM model. Furthermore, the center-of-gravity model and the spatial mismatch index (SMI) were employed to portray the spatial matching characteristics of EE and HSRs. Finally, the DSPDM was constructed to examine the spatial dynamic effect of HSRs on EE and the heterogeneity characteristics.

The study contributes to future research in four ways. First, the multi-source remote sensing data have been combined to enhance the comprehensiveness of the evaluation index system of EE. Second, the spatial distribution pattern and dynamic relationship between EE and HSRs were observed at the county area. After that, the values of the SMI of EE and HSRs were regarded as the basis of heterogeneity in this research. Finally, the quasi-maximum likelihood (QML) estimation method was introduced to estimate DSPDM, which was helpful to reveal the impact of HSRs on EE at different stages. As a result, this ultimately promotes the improvement of EE in general.

## 2. Materials and Methods

### 2.1. Regional Overview

**Figure 1:**
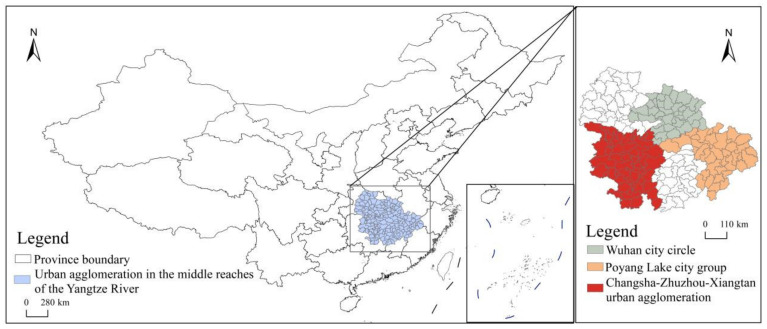
Study Area.

The study area UAMRYR is located in the middle reaches of the Yangtze River economic belt in China, representing an important position for development (Figure 1). According to the *Development Plan of Urban Agglomeration in the Middle Reaches of Yangtze River* (20°09′ N~33°20′ N, 180°21′ E~118°28′ E) approved by the State Council of China in 2015, the UAMRYR includes most areas of Jiangxi, Hubei, and Hunan Provinces, and covers a land area of approximately 317,000 square kilometers. Its topography is mainly alluvial plains with flat terrain. The area has a subtropical monsoon climate, and has many rivers and lakes such as Dongting Lake, Poyang Lake, Han River, and Xiang River. As a result, its water resources are relatively abundant.

### 2.2. Methodology

#### 2.2.1. Evaluation of EE

The Super-SBM model is widely used at present to calculate EE. It was first constructed by Tone and Tsutsui [25], and can make evaluation results more realistic, as shown in Equation (1):

(1) $$\begin{array}{l} \min\rho =\left[1+\frac{1}{m}\sum_{i=1}^{m}s_{i}^{-}/x_{ik}\right]/\left[1-\frac{1}{s}\sum_{r=1}^{s}s_{r}^{+}/y_{rk}\right]\\ \sum_{j=1,j\neq k}^{n}x_{ij}\lambda_{j}-s_{i}^{-}\leq x_{ik}\\ \sum_{j=1,j\neq k}^{n}y_{rj}\lambda_{j}+s_{r}^{+}\geq y_{rk}\\ s_{i}^{-},s_{r}^{+},\lambda_{j}\geq 0 \end{array}$$

where *ρ* denotes the value of EE of each county; $s_{r}^{+}$ and $s_{i}^{-}$ are the expected and non-expected output slack, respectively; *x* and *y* are the input ensemble and output ensemble of EE, respectively; *λ_j_* is the weight vector.

#### 2.2.2. Spatial Matching Features

(1) Center-of-Gravity Model

In this study, the center-of-gravity model was applied to explore the spatial attributes of EE and HSRs [26], as follows:

(2) $$G_{o}\left(x,y\right)=\left[\frac{\sum_{h=1}^{n}\varphi_{oh}\left(e\right)Q_{h}\left(x\right)}{\sum_{h=1}^{n}\varphi_{oh}\left(e\right)},\frac{\sum_{h=1}^{n}\varphi_{oh}\left(e\right)Q_{h}\left(y\right)}{\sum_{h=1}^{n}\varphi_{oh}\left(e\right)}\right]$$

where *φ_oh_*(*e*) represents the value of EE and HSRs, and *Q_h_*(*x*,*y*) is the geographic coordinates of the administrative center of each county.

The model was applied to calculate the interannual center of gravity shift distance, as in Equation (3):

(3) $$D_{i-l}=R\times \sqrt{{\left(y_{i}-y_{l}\right)}^{2}+{\left(x_{i}-x_{l}\right)}^{2}}$$

where *D* is the interannual movement distance of the center of gravity; *R* takes the value of 111.111 km; and (*x_i_*, *y_i_*) and (*x_l_*, *y_l_*) denote the coordinates of the center of gravity in year *i* and year *l*, respectively.

The spatial overlap was measured by the spatial distance between the interannual center of gravity coordinates *G_o_*(*x*,*y*), as follows:

(4) $$S=d_{G_{E}G_{F}}=\sqrt{{\left(x_{E}-x_{F}\right)}^{2}+{\left(y_{E}-y_{F}\right)}^{2}}$$

where *s* denotes the interannual center of gravity of EE and HSRs, and (*x_E_*, *y_E_*) and (*x_F_*, *y_F_*) are the center of gravity coordinates of EE and HSRs, respectively.

The consistency is measures whether the trajectory of the center of gravity change is consistent. The specific calculation formula is shown in Equation (5):

(5) $$V=\cos\theta =\frac{\Delta x_{E}\Delta x_{F}+\Delta y_{E}\Delta y_{F}}{\sqrt{\left(\Delta x_{E}^{2}+\Delta y_{E}^{2}\right)\left(\Delta x_{F}^{2}+\Delta y_{F}^{2}\right)}}$$

where *V* is the consistency index; *θ* denotes the vector angle of the displacement of the center of gravity relative to the previous time point.

(2) Spatial Mismatch Index

This model was introduced to reveal the spatial mismatch posture of EE and HSRs [27]. The formula of the spatial mismatch index is shown in Equation (6):

(6) $$SMI_{i}=\frac{1}{EE}\left(\frac{high_{i}}{high}EE-EE_{i}\right)\times 100$$

where a larger absolute value indicates a lower similarity of spatial distribution, and vice versa. A positive value of *SMI_i_* indicates that the construction of HSRs can fit well with EE, and vice versa.

#### 2.2.3. Spatial Panel Model

EE has been shown to be spatially correlated [28,29,30,31]. Spatial panel models have been widely utilized to explore spatial correlation, spatial spillover, and proximity effects.

(1) Matrix Setting

(1) The geographic distance matrix (W1) is given in Equation (7) [32]:

(7) $$W_{ij}=\left\{\begin{array}{l} 1/d_{ij}^{2},i\neq j\\ 0,i=j \end{array}\right.$$

(2) The economic distance matrix (W2) was constructed as follows (8):

(8) $$\begin{array}{l} W_{ij}=W1\times diag\left(\bar{Y_{1}}/\bar{Y},\bar{Y_{2}}/\bar{Y},\cdots ,\bar{Y_{n}}/\bar{Y}\right)\\ \bar{Y_{i}}=\frac{1}{t_{1}+t_{0}+1}\sum_{t=t_{0}}^{t_{1}}Y_{it},\overset{═}{Y}=\frac{1}{n\left(t_{1}+t_{0}+1\right)}\sum_{i=t_{0}}^{t_{1}}\sum_{i=1}^{n}Y_{it} \end{array}$$

where *W_ij_* is the spatial weight of county *i* and *j*; *W*1 is the geographic distance matrix; $diag\left(\bar{Y_{1}}/\bar{Y},\bar{Y_{2}}/\bar{Y},\cdots ,\bar{Y_{n}}/\bar{Y}\right)$ is the diagonal matrix; ${\bar{Y}}_{i}$ denotes the mean value of real GDP per capita in each year of county *i*; ${\overset{═}{Y}}_{i}$ denotes the mean value of real GDP per capita of the study sample; *t*_0_ is the base period of the study; *t*_1_ is the end of the study.

(2) Spatial Panel Durbin Model (SPDM) and Decomposition

The more generalized SPDM is often selected to measure the spatial effect more comprehensively [33], with the formula given in Equation (9) [34]:

(9) $$Y=\rho WY+X\beta +\theta WX+\alpha l_{n}+\varepsilon$$

where *Y* is the explanatory variable; *ρ* is the spatial autocorrelation coefficient; *X* is the explanatory variable; *W* is the spatial weight matrix; *WY* and *WX* are the spatial lagged terms of the explanatory and explanatory variables; *ɑ* is the constant; *l_n_* is the *n* × 1 unit constant matrix; *β* and *θ* denote the regression coefficients; *ε* is the error term.

The point estimation of the SPDM might be biased [35]. This bias can be overcome by decomposing the total effects into direct and indirect effects, using a partial differential decomposition approach. The formula is given in Equation (10) [36]:

(10) $$Y={\left(I-\rho W\right)}^{-1}\alpha \ln+{\left(I-\rho W\right)}^{-1}(X_{i}\beta +WX_{i}\theta )+{\left(I-\rho W\right)}^{-\varepsilon }$$

(3) Dynamic Spatial Panel Durbin Model (DSPDM) and Decomposition

The DSPDM, which encompasses the spatial lag effects, time lag effects, and temporal double lag effects, was applied to measure the spatial effects. The quasi-maximum likelihood estimation method (BC-QML) was chosen for accurate estimation [37], and the formula is given in Equation (11) [37]:

(11) $$\begin{array}{l} EE_{it}=\alpha +\beta_{1}EE_{i,t-1}+\delta \sum_{i=1}^{n}W_{ij}EE_{jt}+\rho_{1}\sum_{i=1}^{n}W_{ij}EE_{j,t-1}+\beta_{2}HSR_{s,it}\\ +\rho_{2}\sum_{i=1}^{n}W_{ij}HSR_{s,j,t-1}+\theta \sum_{i=1}^{n}X_{it}+\lambda \sum_{i=1}^{n}W_{ij}X_{jt}+\mu_{i}+v_{t}+\varepsilon_{it} \end{array}$$

where *EE_it_* and *EE_i,t−1_* denote the EE in period *t* and period *t*−1 of the *i*th unit, respectively; *HSR_s,it_* denotes the number of HSRs lines opened in period *t* of the *i*th unit; *X_it_* is the ensemble of other control variables; *W* is the spatial weight matrix; *μ* denotes the spatial fixed effects; *ν_t_* denotes the time fixed effects; *ε_it_* is the error term.

### 2.3. Data and Variables

The green development effects of HSRs were the main study goals, and EE represented a proxy variable for the green development effects. Therefore, EE was regarded as the dependent variable in the present research. For consistency with other studies, an evaluation index system of EE based on input-output theory was constructed and is presented in Table 1. Resource input, land input, capital input, and labor input were selected as input indicators, which were expressed as the energy consumption index, construction and arable land areas, fixed asset investment, and total population at the end of the year, respectively. Economic output and environmental output were selected as output indicators, among which GDP was chosen as the desirable output to reflect economic benefit. The annual average PM2.5 concentration was considered the undesirable output, attributed to the lack of data on environmental protection in counties and the existence of more serious similarity of water and air pollution problems in the UAMRYR.

In this study, the number of HSRs (*HSRs*) was the core explanatory variable. The initial value of the unit was assigned as 1 with the presence of a railway station, and the remaining values were assigned as 0. Subsequently opened HSRs were added sequentially on this basis. The data for HSRs was accessed from the China Research Data Service Platform.

The theoretical basis of the control variables is more mature than previous studies. Based on the literature and combined with the availability of county data, seven factors were selected as the control variables:

(1) Economic development (*ECO*). The relationship between economic growth and environment, represented by the environmental Kuznets curve hypothesis, has been abundantly studied [43]. Economy may have two impacts on EE: green effects [44] and black effects [45]. *ECO* is expressed as GDP per capita [46].
(2) Fiscal decentralization (*FD*). The development pattern and efficiency of regional economies are to some extent influenced by the economic policies and allocation of fiscal expenditure set by local government [47], thus EE is influenced by fiscal decentralization. This aspect of the study is in line with the method to calculate *FD* stated by Wu et al. [48].
(3) Industrial structure (*IND*). There are significant differences among industries [31]. industrial structure affects the efficiency of resource allocation, quantities of resource consumption, and the pollution emission status of a region, indirectly affecting the EE [49,50]. In this study, *IND* was measured by dividing the added value of the tertiary sector by the added value of the secondary sector in each county [30].
(4) Population density (*PEO*). While population growth brings dividends to economic development, it also causes environmental pollution [51,52]. Considering the huge population differences among cities in China, the study used population density to reflect the characteristics of population distribution, representing the number of people per unit area within each administrative region [31].
(5) Financial agglomeration (*FIN*). The financial industry is the core of the modern economic system [45]. Scholars have affirmed the importance of financial development in environmental issues [29,53]. Financial agglomeration may indirectly influence EE by acting on regional industrial structure and rational allocation of resources. The *FIN* values were calculated according to the results of Huang et al. [30].
(6) Ecological background (*EB*). The ecological background includes functions such as soil protection and air purification [54], indirectly generating ecological benefits and thus influencing EE. This study uses the NDVI index to represent the ecological background [55,56]. The data used was supplied by the International Scientific Data Mirror Center, Computer Network Information Center, Chinese Academy of Sciences.
(7) Road density (*ROAD*). A better network of transportation is better able to facilitate the flow of production factors [57]. The environmental pollution caused by transportation has also been confirmed to affect EE [2]. In this study, road density was used as a proxy variable, measured by the proportion of total road length to administrative area [31], calculated by the element merge and overlay functions of Arc GIS10.8 software, https://developers.arcgis.com (accessed on 25 January 2021).

It should be noted here that the data without references were sourced from the *China County Statistical Yearbook*, the *China Municipal Statistical Yearbook*, and the *China National Economic and Social Development Statistical Bulletin* for each county.

The collinearity test and unit root test results are shown in Table A1. The missing data were approximated using the mean values. The variance expansion factor of each variable showed that there was no covariance. In addition, the LLC and Fisher-ADF results both passed the significance test, indicating the smoothness of variables.

## 3. Results

### 3.1. Characteristics of Spatial Center of Gravity

**Figure 2:**
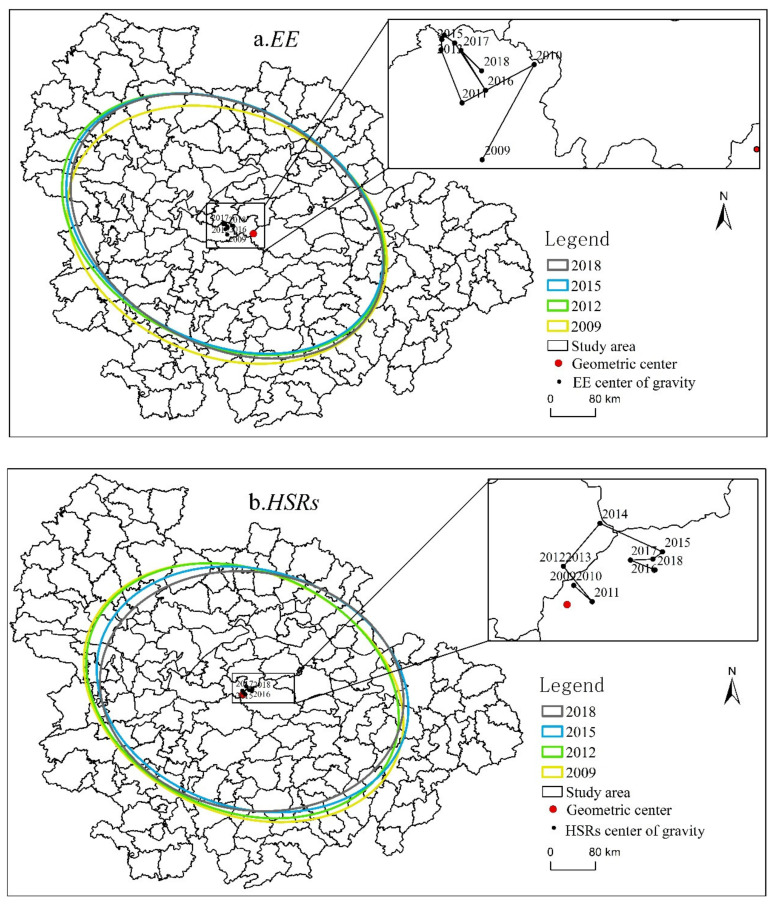
Trajectory of the Centers of Gravity of EE and HSRs (2009–2018).

Table 2 shows the interannual migration distance of the centers of gravity between EE and HSRs from 2009 to 2018. The standard deviation ellipse and the migration trajectory of the centers of gravity are plotted for the selected years 2009, 2012, 2015, and 2018 in Figure 2.

As can be seen from Figure 2, the center of gravity of EE from 2009 to 2018 fell in the northwest of the geometric center of the UAMRYR, indicating that the values of EE in the west and north were greater than those in other regions. The indicators of the standard deviation ellipse for EE suggested that the scale factors were concentrated in the central part of the UAMRYR. The variation characteristics of the elliptic-shaped index and elliptic flatness indicate that the UAMRYR’s production resource factors are in dynamic equilibrium after a certain degree of concentration. The reason is that the northwestern part of the UAMRYR is the Wuhan metropolis, where the overall economic development level is relatively high. The central area of the UAMRYR includes Wuhan city, Changsha city, and Nanchang city. These provincial capital cities will generally gather more factor resources.

In addition, the center of gravity of HSRs was located to the northeast of the geometric center of the UAMRYR, indicating that the number of HSRs opened in the east and north has been larger than that in other regions. From the standard deviation ellipse of HSRs, the construction of HSRs located in the southeast region was shown to have been relatively fast. Its elliptical shape index and elliptical flatness show the gradual expansion of the network and the increase penetration of the HSRs.

In summary, the centers of gravity of EE and HSRs both fell in the same county, and their elliptical parameter values were similar, confirming the fact that the spatial distributions of EE and HSR resources have strong similarities in the UAMRYR.

### 3.2. Characteristics of Spatial Overlap and Consistency

**Figure 3:**
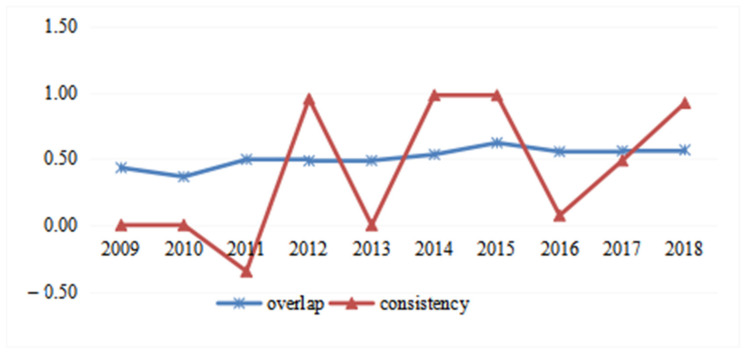
Overlap and Consistency of the Centers of Gravity between EE and HSRs (2009–2018).

As shown in Figure 3, the values of overlap, which fluctuated by approximately 0.500, were from 0.432 in 2009 to 0.566 in 2018, representing an average annual increase of 3.102%, indicating the existence of spatial dislocation between EE and HSRs. In addition, the consistency value was negative in 2011 due mainly to the opening of the first HSRs. The values of consistency reached 1 in the following years, except for was sharp decreases in 2013 and 2016, respectively. These results showed strong spatial synchronization and relatively stable spatial distribution. The sharp decreases of consistency values in 2013 and 2016 can be explained by the launch of a large number of HSRs.

Combining all the results related to the centers of gravity, the spatial distribution between EE and HSRs is similar. The number of HSRs in Wuhan metropolis rank it in first place in the UAMRYR, followed by the Changsha-Zhuzhou-Xiangtan metropolis and Poyang Lake city group. This order is in line with previous research findings [58], and therefore provides a basis for the similarity of the spatial distribution of EE and HSR resources in the UAMRYR.

### 3.3. Spatial Distribution of Spatial Mismatch Index

**Figure 4:**
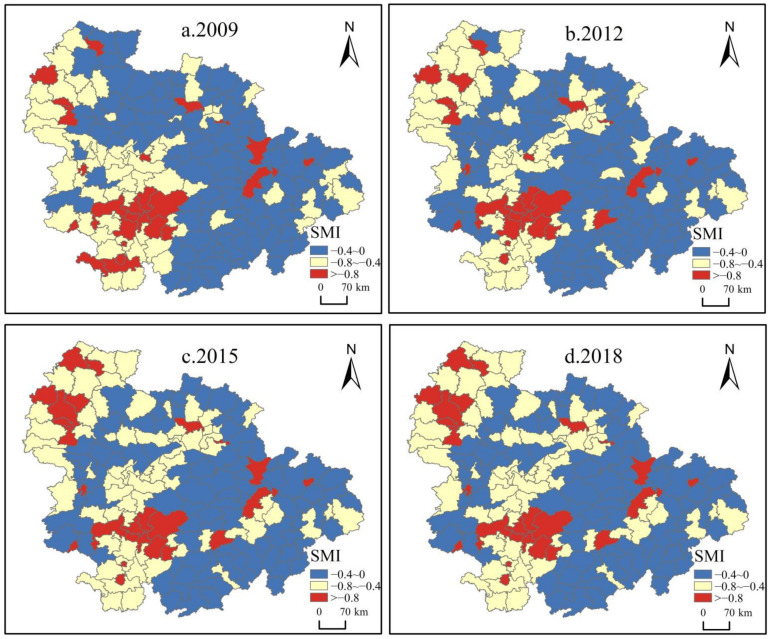
Spatial Mismatch Pattern of EE and HSRs (2009–2018).

Drawing on existing studies [29], the UAMRYR was divided into three areas: negative high spatial mismatch area (SMI < −0.8) (HSMI area), negative medium spatial mismatch area (−0.8 ≤ SMI < −0.4) (MSMI area), and negative low spatial mismatch area (−0.4 ≤ SMI < 0) (LSMI area). The Jenks natural breakpoint method was used for auxiliary expression (Figure 4). It can be observed in Figure 4 that the relationship of EE and HSRs has obvious spatial mismatch features.

From Figure 4, the values of SMI were all less than 0, indicating that the construction of HSRs lagged behind the improvement of *EE*. This is because in the industrial base in the UAMRYR is relatively solid. However, as a modern transportation method, HSRs fail to meet socio-economic developmental needs. Another important reason is that HSRs have a ‘siphon’ effect, leading to regional differentiation.

The gradient pattern of dislocation intensity included dispersal of the HSMI area with the LSMI area, while the values of SMI in the south were lower than those in the north. The spatial distribution of the HSMI area was characterized by a gradual evolution from clustering in the southwest to clustering in the southwest and northwest. Furthermore, an HSMI agglomeration area was formed with the Changsha-Zhuzhou-Xiangtan metropolis and Jingmen-Xiangyang-Yichang city belt as the double core. The spatial distribution pattern of the MSMI area went through a significant process of gathering in the southwest then radiating in the southwest. The reason for this gradual dispersion of the LSMI area and the gradual agglomeration of the HSMI area is that the increasing number of HSRs has indirectly promoted the resource-gathering capacity of certain cities, and the siphoning effect has become more significant, leading to a mismatch between EE and HSRs in more cities.

Overall, the areas with decreased SMI values were mainly concentrated in the southwestern part of the UAMRYR, mostly located in Hunan Province. This is due to the more sophisticated network of HSRs contributing to the harmonious development of the economy and the environment [58]. In contrast, the northern part of the UAMRYR (mainly consisting of Wuhan city, Xiangyang city, and Yichang city) is the dominant region with increased absolute SMI values caused by the mismatch between the network of HSRs and the needs of economic and social development.

### 3.4. Impact of HSRs on EE and Spatial Spillover Effects

#### 3.4.1. Spatial Panel Model Test

Prior to the analysis of the spatial effects, the results of the joint tests were authoritatively considered as the criteria of the model selection. The test results for the four areas are shown in Table 3.

Table 3 shows the coefficient of the Moran index under *W*1, indicating the existence of spatial autocorrelation. The fixed effect was selected according to the results of the Hausman test. According to the results of the joint test, the preferred model of the UAMRYR area under *W*1 was the fixed-effect SPDM. According to these criteria, the fixed-effect SPDM was used for the UAMRYR area under *W*2, and the LSMI, MSMI, and HSMI areas under *W*1. The model of the HSMI area under *W*2 was identified as the fixed-effect SPEM because both the LR test and Wald test had insignificant coefficients, while the coefficients of the LM error and R-LM error tests were statistically significant at the 1% level.

#### 3.4.2. Estimation of the Spatial Spillover Effects

The results of the point estimation are shown in Table A2. Elhorst [37] asserted that the spatial spillover effects calculated in the SPDM are a global effect rather than a local effect. This bias can be effectively solved by decomposing the spatial effect. The results are shown in Table 4.

The values in the first part of the table show the impact of HSRs on local EE (direct effects). Under *W*1, the values indicate that HSRs significantly promoted EE in the four destinations except the HSMI area. This is because most of the units in the HSMI area are municipalities. The large spatial distances lead to weak spatial linkages, which is consistent with the established literature [59]. Under *W*2, green development effects of HSRs are indicated in the UAMRYR and LSMI areas. After considering the economic linkages of the units, the coefficient of the direct effect in the MSMI area was no longer significant. This indicates that the economic development levels of the units vary widely, breaking through the geographical linkages between the units [60]. In summary, the coefficients under the two matrices did not differ greatly, indicating that an economic development mechanism has not yet formed to promote the EE of destinations themselves.

The spatial spillover effects of HSRs on EE (indirect effects) were also determined. Under *W*1, the coefficients were found to be significant in the UAMRYR, LSMI, and MSMI areas, and not significant in the HSMI area. Under *W*2, only the coefficients in the UAMRYR and LSMI area passed the significance level test. Under the two matrices, the situation of the coefficients of the indirect effects was the same as that of the direct effects in all areas, and the reasons were similar. However, the values under *W*2 were all larger than those under *W*1, indicating that the consideration of economic-geographical links between units is more conducive to the establishment of spatial spillover mechanisms.

Finally, the values in the last part of the table show that the composition of direct effects, indirect effects, and total effects was similar under the two matrices. This shows the robustness of the results, and indicates that the impact of HSRs on EE consists mainly of the spatial spillover effects. The above findings can be explained by following two lines of reasoning. First, the mobility of HSRs can link the units into a network. Despite the siphoning effect of this process, it promotes the flow of resources and contributory factors to a certain extent. Second, it reflects the effectiveness of the synergistic development of the UAMRYR, which has formed a spatially linked network and established a spatial spillover mechanism. These findings are consistent with those of previous research [61].

#### 3.4.3. Estimation of the Dynamic Spatial Spillover Effects

The BMQ estimation method was applied to estimate the DSPDM, and the estimation results are presented in Table 5.

As shown in Table 5, the coefficient values of L. ln*EE* and L. W*ln*EE* indicate significant temporal inertia effects of HSRs on EE, and associated spatial dependence characteristics. In other words, the changes in EE were positively influenced by HSRs in the latter period of the study, with a significant spatial diffusion effect. Deng et al. also found that HSRs had a time lag effect [62]. Specifically, this insignificant direct effect shifts from inhibition to promotion, suggesting that HSRs may have a long-term influence in the UAMRYR. In addition, the significant coefficients of indirect SR and LR indicate that the EE of neighboring areas is enhanced by HSRs in both the short and long term.

Additionally, the coefficient values of the indirect short- and long-term effects were fund to be close to the total effects, implying that the effects of HSRs on EE were based mainly on the spatial spillover effects. The coefficient values of the indirect short-term effects were larger than those of the long-term effects. One possible explanation is that in the short term, HSRs opened only in certain some unit areas, which accelerated the resource plundering in these places. With the gradual improvement of the HSR network, unit areas at competitive disadvantage also gradually began to be included in resource allocation, thus this effect of resource plundering gradually diminished.

Finally, the coefficient values for the long-term effects were lower than those of the short-term effects, suggesting an inverted V-shaped relationship between EE and HSRs during the study period. This may be due to several reasons. First of all, when the number of HSRs increases to the point of saturation or even redundancy of market demand, oversupply leads to low economic returns and high energy consumption. Furthermore, the expansion of the output scale brought by the HSRs counteracts the effects of emission reductions by increasing pollution emissions, which eventually has a negative impact on the environment, as confirmed by Chen et al. [63].

## 4. Discussion

### 4.1. Discussion of the content

Currently, the construction of an ecological civilization in the UAMRYR in China is in a period of accelerated transformation, and HSRs play an important role in affecting EE. The results of this study are helpful to enrich the theoretical system of green development from the perspective of EE. The study can help the government and various departments to understand the spatial relationship of EE and HSRs, identify spatial matching of EE and HSRs, and determine the long- and short-term differences and heterogeneity of the effects of HSRs on EE.

In this study, based on previous research, the green development effects of HSRs have been explored, instead of their single economic or environmental effects. Furthermore, the spatial matching characteristics of HSRs and EE have been portrayed, and the heterogeneous differences in the long- and short-term green development effects of HSRs revealed. In summary, this study refines the established literature in terms of research perspective, content, and methodology.

The results of the study suggest that the spatial distributions of EE and HSRs are similar. However, spatial misalignment characteristics indicate that in the UAMRYR, the construction of HSRs still trails the improvement of EE. Therefore, this similarity and dislocation need to be considered simultaneously to improve EE in the UAMRYR from the perspective of HSRs. As an advancement of the established research, the existence was identified of spatial spillover of the green development effects of HSR. In this study, the green development effects of HSR in each of the four regions were explored. Except for the HSMI region, all others showed the green development effects of HSR, which demonstrates the value of studying the green development effects brought by HSRs. Furthermore, this study found that the effects of HSRs on EE in the UAMRYR are based mainly on spatial spillover effects, where the effects are stronger and more obvious in the short term. This finding provides implications for future adjustments of HSRs. To conclude, this study provides new perspectives for other regions to enhance green development associated with HSRs.

### 4.2. Policy Implications

Based on the above analysis, the current study identifies three specific implications for policy makers:

(1) Fully evaluate and utilize the external effects brought about by HSRs. In the UAMRYR, the network of HSRs has been gradually improved, but the presence of spatial agglomerations is significant. Some cities have been affected by siphon effects. Therefore, HSRs should be planned reasonably in accordance with the specific development situations of cities, focusing particularly on solving the problem of redundancy of HSR resources in station cities with little demand for HSRs, in order to sustain the contribution of HSRs to EE.
(2) Focus on the HSMI areas between EE and HSRs. The values of SMI have obvious regional differences, which should be fully considered when allocating resources of EE and HSRs. Specifically, the formation mechanism of the HSMI area can be clarified by means of geography and statistics, to continuously play the driving role of the core city circle, improve the diffusion effects on peripheral cities, and promote the overall spillover of EE.
(3) Construct a model reflecting the direct effects of HSRs on EE. In this study, the short-term effects of HSRs on the EE of the UAMRYR were found to be more significant than the long-term effects. The total effects were mainly the results of spillover. Therefore, future development strategies should be based on maintaining the structure of urban clusters, optimizing the allocation of resources on a larger scale, strengthening the construction of urban network diffusion, and thus establishing a mechanism for the benign effects of HSRs on EE.

### 4.3. Limitations and Future Directions

As a preliminary exploration of the dynamic relationship between HSRs and EE, this study is not free from limitations. Future studies could explore the relationship between HSRs and EE from the following aspects. First, the mechanism path of HSRs on EE should be tested. The mediating effect model and the construction of an interaction term can be used to examine more deeply the paths and mechanisms of HSRs’ effect on EE. Second, the nonlinear character of the relation between HSRs and EE should be examined. Linkages between regions are complex and dynamic and often have nonlinear characteristics. Therefore, nonlinear parameter estimation instruments, such as panel threshold models and panel smoothed transformation models, need to be utilized to explore the nonlinear effect of HSRs on EE. Third, the nature of the effects of HSRs on EE should be estimated. The use of VAR models and machine learning should be considered in future studies to clarify the specific intervals and inflection points of the effects of HSRs on EE.

## 5. Conclusions

Regarding the spatial and temporal patterns of EE and HSRs in the UAMRYR, the coordinates of the interannual centers of gravity of EE and HSRs fall in the same county. The values of each standard deviation ellipse parameter of EE and HSRs are very close, indicating the similarity of the spatial distribution of EE production factors and HSR resources.

In terms of spatial matching between EE and HSRs, there are significant spatial mismatch characteristics and negative spatial mismatch indices, indicating that the construction of HSRs has lagged behind the improvement of EE. Furthermore, the spatial distribution pattern of the LSMI area was found to be relatively concentrated, the HSMI area was randomly distributed, the MSMI and LSMI areas were interspersed, and the dislocation intensity had the gradient pattern of low value in the south and high value in the north.

Observing the spatial effects of HSRs on EE, the coefficients under the two spatial matrices in the UAMRYR and the LSMI area were found to be significantly positive. The coefficients in the MSMI area were significant under *W*1 and insignificant under *W*2, the coefficient of the HSMI area was not significant because it consists mainly of municipal districts, and the spatial fragmentation leads to weak intercounty connectivity

The estimation results of the DSPDM showed that the path-dependent characteristics and spatial spillover effects of the dynamic impact of HSRs on EE in the UAMRYR. In both the long-term and short-term estimation results, the indirect effect was found to be the main source of the total effects, and the impact of HSRs on EE was dominated by the spatial spillover effects. In addition, the coefficient values of the short-term effects were significantly larger than those of the long-term effects, which means that the effects of HSRs on EE are stronger and more obvious in the short term.

## Figures and Tables

**Table 1:** Evaluation Index System of EE.

Type	Indicator (Reference)	Data Type	Data Source	Data Processing
Inputs	Energy consumption index [38]	DMSP/OLS night light data	Data center for resource and environmental sciences, Chinese Academy of Sciences	Partition statistics summation in Arc GIS 10.8
	Construction site area [39]	Remote sensing monitoring data of land use status in China	Geographical state monitoring cloud platform	Area tabulation in Arc GIS10.8
	Arable land area [39]			
	Total social fixed asset investment (10,000 yuan) [40,41]	Statistic data	Chinese county statistical yearbooks, statistical yearbook of each city, statistical bulletin of national economic and social development of each district and county	—
	Total population (10,000 people) [28]	Statistic data		—
Desirable output	GDP (10,000 yuan) [38]	Statistic data		Deflating data using 2009 as the base period
Indesirable output	Annual average concentration of PM2.5 [29,42]	Aerosol inversion remote sensing data	Atmospheric composition analysis organization	Partition statistics in Arc GIS10.8 to find the mean value

**Table 2:** Characteristics of the Centers of Gravity of EE and HSRs (2009–2018).

Time	2009	2010	2011	2012	2013	2014	2015	2016	2017	2018
EE-A	—	45.214	39.216	32.835	13.858	9.654	15.712	32.631	29.682	23.344
EE-B	2.648	—	—	2.727	—	—	2.683	—	—	2.682
EE-C	1.963	—	—	1.918	—	—	1.906	—	—	1.905
EE-D	0.741	—	—	0.704	—	—	0.710	—	—	0.710
EE-E	0.685	—	—	0.809	—	—	0.777	—	—	0.777
EE-F	112.047	—	—	116.309	—	—	117.053	—	—	119.862
HSR-A	—	0.000	22.205	30.226	0.000	33.640	37.141	15.515	21.231	23.005
HSR-B	2.669	—	—	2.700	—	—	2.549	—	—	2.449
HSR-C	1.943	—	—	1.932	—	—	1.913	—	—	1.912
HSR-D	0.728	—	—	0.743	—	—	0.751	—	—	0.781
HSR-E	0.726	—	—	0.668	—	—	0.636	—	—	0.538
HSR-F	113.752	—	—	113.121	—	—	116.967	—	—	99.376

Note: A = migration distance of center of gravity; B = long axis of the ellipse; C = short axis of the ellipse; D = oval-shaped index = elliptical short axis-elliptical long axis; E = elliptic flat rate = elliptical long axis-elliptical short axis; F = ellipse corner; the unit of ellipse corner is ‘°’; the unit of elliptic flat rate is ‘—’; remaining index unit is ‘km’.

**Table 3:** Results of Spatial Panel Model Test.

Region	Matrix	Moran’s I	Hausman	LR- Spatial	LR- Time	Wald- SLM	Wald- SEM	LR- SLM	LR- SEM	LM- Lag	LM- Error
UAMRYA area	*W*1	503.19 **	34.60 ***	12.02	3137.66 ***	46.33 ***	55.04 ***	45.95 ***	54.76 ***	1.33 (3.588 *)	15.17 *** (17.43 ***)
	*W*2	96.36 ***	−200.21	27.54 ***	3448.18 ***	81.19 ***	92.36 ***	89.94 ***	97.31 ***	0.01 (1.41)	68.96 *** (70.36 **)
LSMI area	*W*1	1539.30 ***	67.18 ***	17.65 *	1058.53 ***	14.14 *	14.36 *	16.05 *	14.31 *	0.31 (0.02)	38.13 *** (37.84 ***)
	*W*2	441.18 ***	113.70 ***	13.88	1082.36 ***	17.56 **	17.56 **	17.48 **	17.15 **	0.81 (0.15)	150.93 *** (150.26 ***)
MSMI area	*W*1	1529.34 ***	−51.42	8.49	956.88 ***	13.57 *	42.18 ***	13.85 *	42.33 ***	0.04 (0.15)	27.74 *** (27.86 ***)
	*W*2	251.53 ***	−16.23	25.20 ***	923.97 ***	81.86 ***	123.90 ***	90.00 ***	116.74 ***	0.01 (0.95)	80.95 *** (81.89 **)
HSMI area	*W*1	1650.26 ***	15.82 **	24.21 ***	460.38 ***	18.03 **	18.05 **	17.37 **	17.35 **	0.23 (1.83)	5.47 ** (7.08 ***)
	*W*2	190.59 ***	19.04 **	25.57 ***	460.16 ***	13.30	12.87	12.92	12.47	0.27 (2.35)	26.49 *** (28.56 ***)

Notes: ***, **, * are statistically significant at 1%, 5%, and 10%, respectively. The significance of all subsequent tables is aligned with this. The corresponding robustness test results are shown in parentheses.

**Table 4:** Decomposition Results of the Spatial Panel Model.

Area	UAMRYR Area		LSMI Area		MSMI Area		HSMI Area
Matrix	*W*1	*W*2	*W*1	*W*2	*W*1	*W*2	*W*1
Variables	Direct effects						
ln*HSRs*	0.038 ** (2.45)	0.048 *** (3.09)	0.046 * (1.74)	0.048 * (1.82)	0.026 * (1.81)	0.018 (1.17)	0.076 (1.62)
ln*ECO*	0.401 *** (10.76)	0.396 *** (10.60)	0.092 (1.60)	0.034 (0.59)	0.458 *** (10.12)	0.493 *** (9.30)	0.848 *** (7.18)
ln*FD*	−0.041 *	−0.038 * (−1.67)	−0.003 (−0.09)	−0.013 (−0.46)	0.011 (0.37)	0.042 (1.26)	−0.113 (−1.34)
	(−1.79)						
ln*IND*	−0.009 (1.58)	−0.015 (−0.85)	0.012 (0.45)	−0.004 (−0.16)	−0.004 (−0.18)	−0.028 (−1.14)	−0.123 ** (−2.34)
ln*PEO*	0.079 (1.58)	0.071 (1.43)	−0.071 (−0.85)	−0.174 ** (−2.00)	0.438 *** (7.63)	0.598 *** (6.66)	0.212 (1.64)
ln*FIN*	0.011 (1.32)	0.014 * (1.68)	0.002 (0.29)	0.002 (0.29)	0.076 *** (4.32)	0.132 *** (4.35)	0.063 (1.13)
ln*EB*	−0.001 (−0.65)	−0.001 (−0.73)	−0.001 (−0.47)	−0.001 (−0.34)	0.001 (0.26)	0.001 (0.32)	−0.006 (−0.98)
ln*ROAD*	0.055 ** (2.07)	0.044 * (1.65)	0.025 (0.72)	0.037 (0.97)	−0.034 (−1.06)	−0.046 (−1.05)	0.298 ** (2.23)
	Indirect effects (spatial spillover effects)						
ln*HSRs*	0.299 *** (3.33)	0.503 *** (2.98)	0.290 *** (2.63)	0.507 *** (2.84)	0.174 * (1.92)	0.249 (0.45)	0.045 (0.47)
ln*ECO*	0.564 ** (2.11)	2.215 *** (3.39)	0.219 *** (3.10)	−1.352 ** (−2.21)	1.005 *** (2.63)	3.712 * (1.89)	0.001 (0.01)
ln*FD*	0.207 (1.29)	0.21 (0.73)	0.124 (1.03)	−0.266 (−1.09)	−0.107 (−0.37)	0.48 (0.46)	0.194 (0.77)
ln*IND*	−0.063 (−0.60)	0.213 (0.90)	0.018 (0.26)	−0.074 (−0.61)	−0.137 (−0.83)	−1.535 ** (−2.02)	0.127 (1.24)
ln*PEO*	0.582 * (1.71)	2.042 ** (2.19)	0.118 (0.42)	−0.715 (−1.15)	1.455 *** (2.89)	10.400 ** (2.32)	0.946 *** (−2.80)
ln*FIN*	0.152 ** (2.26)	0.859 *** (2.78)	0.012 (0.41)	0.071 (1.04)	0.472 ** (2.29)	3.634 ** (2.34)	0.441 * (1.90)
ln*EB*	−0.001 (−0.12)	−0.013 (−0.65)	0.006 (0.88)	0.011 (0.86)	0.025 (1.47)	0.061 (0.89)	−0.007 (−0.47)
ln*ROAD*	−0.973 *** (−3.49)	−1.196 *** (−2.65)	−0.125 (−1.10)	0.038 (0.08)	−0.331 (−0.96)	−1.449 (−0.85)	0.389 (1.06)
	Total effects						
ln*HSRs*	0.337 *** (3.85)	0.551 *** (3.31)	0.336 *** (2.96)	0.555 *** (3.09)	0.200 ** (2.12)	0.166 (0.50)	0.121 (1.34)
ln*ECO*	0.965 *** (3.55)	2.611 *** (3.99)	0.311 *** (7.56)	−1.318 ** (−2.15)	1.463 *** (3.61)	4.205 ** (2.10)	0.849 *** (2.79)
ln*FD*	0.165 (0.97)	0.172 (0.66)	0.122 (0.96)	−0.279 (−1.11)	−0.096 (−0.31)	0.522 (0.49)	0.081 (0.30)
ln*IND*	−0.072 (−0.68)	0.198 (0.84)	0.029 (0.49)	−0.078 (−0.68)	−0.141 (−0.81)	−1.563 ** (−2.03)	0.004 (0.04)
ln*PEO*	0.661 * (1.83)	2.114 ** (2.23)	0.047 (0.15)	−0.889 (−1.34)	1.893 *** (3.54)	10.998 ** 92.41)	−0.734 ** (−2.07)
ln*FIN*	0.163 ** (2.35)	0.873 *** (2.81)	0.014 (0.45)	0.073 (1.08)	0.548 ** (2.54)	3.765 ** (2.39)	0.504 ** (2.05)
ln*EB*	−0.002 (−0.22)	−0.014 (−0.71)	0.005 (0.73)	0.01 (0.81)	0.025 (1.41)	0.061 (0.89)	−0.013 (−0.77)
ln*ROAD*	−0.918 *** (−3.18)	−2.151 ** (−2.57)	−0.100 (−0.88)	0.075 (0.16)	−0.364 (−0.99)	−1.496 (−0.86)	0.687 (1.64)

Notes: ***, **, * are statistically significant at 1%, 5%, and 10%, respectively. *t* values are shown in parentheses. The significance of all subsequent tables is aligned with this.

**Table 5:** Results of the DSPDM.

		*Wx*	SR-Direct	SR-Indirect	SR-Total	LR-Direct	LR-Indirect	LR-Total
L.ln*EE*	0.453 *** (20.42)							
ln*HSRs*	0.009 (0.59)	−0.444 *** (−4.82)	−0.004 (−0.06)	0.391 *** (3.38)	0.386 *** (4.21)	0.031 (1.36)	0.056 * (2.26)	0.087 *** (5.22)
ln*ECO*	0.225 *** (5.40)	−4.165 *** (−9.95)	0.093 (0.17)	3.337 *** (4.43)	3.431 *** (6.24)	0.476 *** (7.00)	0.298 ** (3.17)	0.774 *** (9.32)
ln*FD*	0.006 (0.24)	1.701 *** (8.59)	0.067 (0.26)	−1.559 *** (−4.18)	−1.493 *** (−5.15)	−0.520 (−1.22)	−0.283 *** (−5.22)	−0.335 *** (−8.26)
ln*IND*	−0.019 (−1.06)	0.546 *** (4.16)	−0.001 (−0.01)	−0.471 ** (−2.90)	−0.471 *** (−3.41)	−0.046 (−1.73)	−0.060 (−1.76)	−0.106 *** (−3.96)
ln*PEO*	−0.040 (−0.74)	0.509 (0.90)	−0.029 (−0.25)	−0.367 (−0.66)	−0.396 (−0.77)	−0.076 (−0.97)	−0.012 (−0.08)	−0.088 (−0.77)
ln*FIN*	−0.007 (−0.95)	−0.467 ** (−3.05)	−0.026 (−0.33)	0.444 ** (2.61)	0.418 ** (2.81)	0.006 (0.38)	0.088 ** (3.08)	0.094 ** (3.07)
ln*EB*	−0.001 (−0.07)	0.005 (0.35)	0.001 (0.09)	−0.005 (−0.39)	−0.005 (−0.39)	−0.001 (−0.06)	−0.001 (−0.32)	−0.001 (−0.40)
ln*ROAD*	0.071 ** (2.87)	−1.077 ** (−3.11)	0.037 (0.24)	0.847 * (2.27)	0.884 ** (2.73)	0.144 *** (3.87)	0.055 (0.74)	0.200 ** (2.88)
L.W*ln*EE*	3.442 *** (16.77)							
ρ	2.160 *** (12.60)							
R2	0.184							
Log-L	1217.9							

Notes: ***, **, * are statistically significant at 1%, 5%, and 10%, respectively. *t* values are shown in parentheses. The significance of all subsequent tables is aligned with this.

## Data Availability

Not applicable.

## Appendix A

**Table A1:** Collinearity Test and Unit Toot Test.

Test	ln*EE*	ln*HSRs*	ln*ECO*	ln*FD*	ln*IND*	ln*PEO*	ln*FIN*	ln*EB*	ln*ROAD*
Co-linearity test	—	1.282	1.826	1.493	1.078	1.863	2.370	1.025	1.014
LLC test	−76.188 ***	−19.086 ***	−59.501 ***	−33.937 ***	−26.592 ***	−472.965 ***	−94.973 ***	−46.044 ***	−16.793 ***
Fisher-ADF test	739.259 ***	157.926 ***	937.797 ***	584.222 ***	501.558 ***	850.320 ***	1259.83 ***	783.086 ***	758.650 ***

Note: *** indicate 1% significance levels.

**Table A2:** Results of Spatial Panel Model.

Variables	Whole Area		LSMI Area		MSMI Area		HSMI Area	
	*W*1	*W*2	*W*1	*W*2	*W*1	*W*2	*W*1	*W*2
ln*HSRs*	0.033 ** (2.10)	0.046 *** (3.02)	0.042 (1.64)	0.050 ** (1.96)	0.019 (1.36)	0.015 (1.02)	0.075 * (1.65)	0.081 ** (2.01)
ln*ECO*	0.393 *** (10.17)	0.1391 *** (10.07)	0.092 (1.54)	0.028 (0.48)	0.420 *** (9.97)	0.441 *** (9.83)	0.852 *** (6.99)	0.797 *** (7.25)
ln*FD*	−0.047 ** (−2.03)	−0.041 * (−1.74)	−0.007 (−0.22)	−0.018 (−0.59)	0.012 (0.49)	0.032 (1.18)	−0.121 (−1.37)	−0.103 (−1.15)
ln*IND*	−0.007 (−0.39)	−0.015 (−0.83)	0.012 (0.44)	−0.004 (−0.16)	0.002 (0.12)	−0.005 (−0.22)	−0.122 ** (−2.28)	−0.136 *** (−2.67)
ln*PEO*	0.070 (1.44)	0.066 (1.32)	−0.072 (−0.86)	−0.179 ** (−1.99)	0.382 *** (7.55)	0.447 *** (8.28)	0.207 (1.63)	0.234 ** (2.00)
ln*FIN*	0.008 (0.97)	0.011 (1.33)	0.001 (0.13)	0.002 (0.28)	0.057 *** (3.65)	0.078 *** (4.64)	0.063 (1.10)	0.044 (0.75)
ln*EB*	−0.001 (−0.66)	−0.001 (−0.73)	−0.001 (−0.52)	−0.001 (−0.32)	−0.001 (−0.31)	0.001 (−0.12)	−0.006 (−1.04)	−0.003 (−0.61)
ln*ROAD*	0.072 *** (2.74)	0.052 * (1.93)	0.027 (0.77)	0.039 (0.98)	−0.020 (−0.79)	−0.024 (−0.84)	0.300 ** (2.18)	0.241 * (1.83)
*W* *ln*HSRs*	0.144 *** (2.86)	0.312 *** (3.36)	0.230 ** (2.44)	0.647 *** (2.95)	0.035 (1.22)	0.018 (0.25)	0.043 (0.42)	
*W* *ln*ECO*	0.118 (0.77)	1.329 *** (3.53)	0.169 ** (2.27)	−1.753 ** (−2.31)	−0.021 (−0.21)	0.464 (1.59)	0.014 (0.04)	
*W* *ln*FD*	0.135 (1.59)	0.157 (0.87)	0.113 (1.11)	−0.339 (−1.1)	−0.039 (−0.49)	0.087 (0.40)	0.211 (0.81)	
*W* *ln*IND*	−0.038 (−0.66)	0.127 (0.91)	0.009 (0.17)	−0.111 (−0.77)	−0.046 (−1.04)	−0.350 *** (−2.93)	0.120 (1.21)	
*W* *ln*PEO*	0.290 (1.57)	1.344 *** (2.71)	0.132 (0.55)	−0.942 (−1.13)	0.142 (1.12)	1.948 *** (4.75)	−0.950 *** (−2.69)	
*W* *ln*FIN*	0.077 ** (2.27)	0.557 *** (3.99)	0.009 (0.36)	0.085 (1.04)	0.091 * (1.73)	0.733 *** (4.60)	0.441 ** (1.97)	
*W* *ln*EB*	0.010 (−0.01)	−0.008 (−0.57)	0.005 (0.94)	0.014 (0.88)	0.007 (1.60)	0.014 (0.97)	−0.007 (−0.47)	
*W* *ln*ROAD*	−0.559 *** (−4.22)	−1.444 *** (−3.86)	−0.119 (−1.32)	0.078 (0.13)	−0.008 (−0.91)	−0.301 (−0.89)	0.410 (1.08)	
*ρ*	0.455 *** (7.91)	0.300 ** (2.08)	0.160 ** (2.09)	−0.337 * (−1.76)	0.716 *** (15.63)	0.755 *** (10.94)	−0.050 (−0.38)	
*λ*								−0.215 (−0.92)
R2	0.467	0.288	0.273	0.178	0.360	0.495	0.315	0.262
Log-L	1139.953	1129.905	627.519	637.793	720.488	701.013	109.903	101.659
Space fixed	yes	yes	yes	yes	yes	yes	yes	yes
Time fixed	yes	yes	yes	yes	yes	yes	yes	yes

Note: ***, **, * are statistically significant at 1%, 5%, and 10%, respectively. The significance of all subsequent tables is aligned with this. *t* values are shown in parentheses. All subsequent tables are aligned with this.
